# Characteristics on the oxidation stability of infant formula powder with different ingredients during storage

**DOI:** 10.1002/fsn3.1928

**Published:** 2020-10-10

**Authors:** Weijun Wang, Yanhua Li, Liqin Cai, Luping Fang

**Affiliations:** ^1^ Research & Development Institute Zhejiang Yi Ming Food Co. Ltd. Wenzhou China; ^2^ College of Food Science and Biotechnology Engineering Zhejiang Gongshang University Hangzhou China

**Keywords:** different ingredients, infant formula powder, oxidation stability, storage

## Abstract

Infant formula powder is prone to oxidation reaction during storage, which leads to the decrease of milk powder quality. The whole milk powder (WMP) was formulated, and the characteristics of infant formula powder were tracked during storage. The addition of metal ions, polyunsaturated fatty acids, and vitamins could reduce the peroxide value and increase the thiobarbituric acid value in the infant formula powder during the early stage of storage. When the samples were stored for 6 months, the free fat content of the base milk powder and the sample added with metal ions had high level (3.3%–3.6%). With adding vitamins, the content of free fat in the samples decreased first and then increased. The color value L of all the samples decreased during storage. Compared with WMP, the color value B of all the infant formula powder with different ingredients decreased. Levels of 2‐heptanone and 2‐nonaone indicated that the formation of the main methyl ketones in the infant formula powder with different ingredients decreased. The content of hexanal in the sample added metal ions was the highest. The type and intensity of free radicals changed with the formula components. The range of g value was 2.0043–2.0060 after 6 months of storage and 2.0017–2.1338 after 12 months of storage, respectively. The index of peroxide value and color value B were significantly related to the existence of free radicals in the infant formula powder with different ingredients.

## Graphical abstract

Changes in the peroxide value, thiobarbituric acid value, free fat content, color value, oxidation flavor compounds, and free radicals were investigated to estimate the oxidation stability of milk powder with different ingredients.

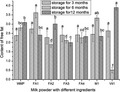

**Practical applications**

Understanding the oxidation stability is important for improving the quality of dairy products. In this study, changes in the peroxide value, thiobarbituric acid value, free fat content, color value, oxidation flavor compounds, and free radicals were investigated to estimate the oxidation stability of infant formula powder with different ingredients. The indicators related to the oxidation stability of infant formula powder with different ingredients changed differently during storage. Additionally, the presence of free radicals was irregular. The type and intensity of free radicals changed with the formula components.

## 1 INTRODUCTION

Milk powder is a favorite food for consumers. However, milk powder is prone to lipid oxidation during processing and storage. Free radicals are produced in the induction stage of lipid oxidation. Then hydroperoxides are formed from free radicals by reaction with oxygen (Hong et al., 2017). That will automatically form chain reaction and promote some related reactions, leading to the decline of milk powder quality (Cluskey et al., 2006; Concetta et al., 2020; Nursten, 1997). The oxidation deterioration during the processing and storage of milk powder is the main bottleneck of its consumption and application (Hough et al., 2002). The oxidation of milk powder is affected by factors, such as processing technology, the compositions, and storage conditions (Cluskey et al., 2006; Li, Zhang, Wang, Han 2013; Vignolles et al., 2007). According to the needs of consumers, it is necessary to mix different components to prepare infant formula powder (Hong et al., 2019; Jeske et al., 2019). Infant formula powder is rich in fat, protein, carbohydrate, and other components, which could influence the lipid oxidation during storage.

The oxidation of formula milk powder has attracted the attention of researchers (Concetta et al., 2020; Romeu‐Nadal et al., 2007). The most reported problem is the oxidation of milk powder caused by the adjustment of unsaturated fatty acids (Romeu‐Nadal et al., 2007). In the infant formula powder, it is most common to adjust the proportion of unsaturated fatty acids by mixing docosahexaenoic acid (DHA) or arachidonic acid (AA). These polyunsaturated fatty acids are easily oxidized to secondary lipid oxidation products (Hong et al., 2019). It was shown that the higher the unsaturation is, the easier it will be oxidized (Parker et al., 2003). Rodriguez‐Alcala et al. (2007) found that the content of monounsaturated fatty acids and polyunsaturated fatty acids in infant formula powder decreased gradually with the prolongation of the storage period. A high peroxide value can be produced by lipid oxidation in infant formulas powder (Cesa et al., 2012; Romeu‐Nadal et al., 2007). Avoiding the contact between oxygen and milk powder could delay the oxidation of milk fat. Antioxidant is the substance that could delay or prevent lipid oxidation, including free radical absorbers, oxygen scavengers, and metal ion chelators. The presence of vitamin C, vitamin E, and other antioxidants in infant formula powder could slow down the oxidation of fat. But high concentration of antioxidants may lose its antioxidant capacity and even promote oxidation promoting capacity (Zou et al., 2015).

The presence of metal ions in milk powder could accelerate the oxidation of milk fat. It has long been recognized that copper and iron are the principal metals involved. These ions function as pro‐oxidants primarily by decomposing hydroperoxides to generate new reaction chains (O’Brien & O’Connor, 2011). Infant formula powder stored at low temperature shows good oxidation stability (Romeu‐Nadal et al., 2007). The oxidation rate of milk fat in WMP, which is stored at low temperature, is much slower than that at high temperature. High temperature will accelerate the oxidation rate of milk fat and the decomposition rate of hydroperoxides in milk powder (Stapelfeldt et al., 1997). It also noted that the level of free radicals in low‐heated milk powder was high after storage at 45°C for 47 days, and there were no significant differences in the sensory scores among milk powder with different water activities. The lipid oxidation of milk powder with different ingredients mainly results from the presence of fat, unsaturated fatty acids, oxidants, and antioxidants. Although it is commonly used to control the oxidation of infant formula powder by controlling water activity, reducing storage temperature and adjusting oxygen concentration (Hong et al., 2019; Lai & Paterson, 2020; Turner et al., 2002), oxidation remains a problem in the quality reduction of milk powder during storage. It is certain that it will lead to the chain reaction of lipids as long as there are free radicals. For the control of oxidation reaction and the improvement of quality of infant formula powder with different ingredients, it is of great significance to study the change of oxidation stability index and its relationship with free radical during the storage of milk powder.

## 2 MATERIALS AND METHODS

### 2.1 Formula design of milk powder sample

Whole milk powder was taken sampled after manufacture. The average contents of the components in the WMP were as follows: protein, 26.5 ± 0.8%; fat, 26.7 ± 1.7%; humidity, 3.0 ± 0.4%. The ingredients in the infant formula powder were made of food grade. The base powder (labeled FA1) was used to prepare other infant formula powder with different ingredients, which was mixed with WMP as following: WMP (1.5 kg), desalted whey powder (3 kg), lactose (0.5 kg) and nondairy cream (3 kg). The content of DHA in FA2 was 0.5 g/kg, and the content of AA in FA3 was 1.25 g/kg. The content of DHA and AA in FA4 was 0.5 g/kg and 1.25 g/kg, respectively. M1 was prepared with FA4, and the content of Zn, Fe, Cu, and Mn was controlled 62.5, 62.5, 3.75, and 3.75 mg/kg, respectively. Vit1 was prepared with FA4, and the content of L‐ascorbic acid and vitamin E was controlled 2.5 and 0.125 g/kg, respectively. The nondairy cream was made by spray drying, which was composed of vegetable oil, glucose syrup, sodium caseinate and emulsifier. The content of surface fat, total fat, and protein was 0.75 ± 0.02%, 24.42 ± 0.08%, and 1.10 ± 0.04%, respectively. Milk powder sample was placed in separated packages and stored hermetically at room temperature.

### 2.2 Determination of the peroxide value

The peroxide value (POV) was determined according to the method described previously (Zou, 2002). Milk powder sample (5.0 g) was mixed with chloroform‐glacial acetic acid solution (40:60, v/v, 5 ml), and saturated potassium iodide solution (1 ml) was added. The mixture was reacted in the dark for 3 min. Then, it was diluted with distilled water (50 ml) and added to starch solution (1%, w/v, 1 ml). The clear liquor was separated by filtration, and the POV was determined at 585 nm.

### 2.3 Determination of the thiobarbituric acid value

The thiobarbituric acid (TBA) value was determined according to the method described previously (Sun, 2013). Milk powder sample was reconstituted (12%, w/v), and the reconstituted milk (35.2 ml) was kept in a water bath (30°C). Then, trichloroacetic acid solution (40%, w/v, 2 ml), and ethanol solution (95%, 4 ml) were added. After shaking, the mixture was allowed to rest for 15 min. Then, the milk fat and proteins were removed by filtration (with qualitative filter paper). TBA solution (0.1 mol/L, 1.0 ml) was added to the clarified filtrate, and the mixture was incubated in a water bath (60°C) for 60 min. The absorbance was measured at 538 nm at room temperature.

### 2.4 Determination of free fat on the surface

Milk powder sample (5.0 g) was added to petroleum ether (50 ml, the boiling point was 30–60°C) and left to stand for 12 hr. The sample was sealed immediately, and the free fat dissolved at room temperature over 15 min. The content of free fat was determined after evaporation (Li et al., 2019).

### 2.5 Determination of the color value

The color change mainly provides the information about Maillard reaction in milk powder. In this study, the differences in the color values among the samples were measured using a colorimeter (Chroma Meter CR‐400, Keshenghang). The color parameters L and B values were analyzed to compare the color changes in different infant formula powder. The L value represents brightness, L = 0 is defined as black, and L = 100 is as white. The B value represents yellowness to blueness, a positive value represent yellowness.

### 2.6 Determination of oxidation flavor compounds

The volatiles in the reconstituted milk powder were extracted and analyzed using head space solid‐phase microextraction‐gas chromatography‐mass spectrometry (HS‐SPME‐GC‐MS) according to a previous study (Li, Zhang, Wang, 2013). The volatiles of hexanal, 2‐heptanone and 2‐nonanone, as the selected oxidized volatiles, were identified by comparison to NIST‐02L GC‐MS spectrum library and the retention time of their standard chemicals (Sigma, USA).

### 2.7 Free radicals in infant formula powder during storage

The radical was measured by an electron spin resonance (ESR) spectrometer (Bruker BioSpin GmbH, Germany). ESR analysis was conducted based on the method described by Thomsen et al. (2005a), Thomsen et al. (2005b). The following instrument parameters were used: sweep width, 100 Gauss; microwave power, 3.10 mW; modulation amplitude, 1.00 Gauss; receiver gain, 5.02 × 10^4^; time constant, 40.96 ms; conversion time, 160 ms; and total sweep time, 163.84 s.

### 2.8 Statistical analysis

Experimental data were statistically analyzed using PASW Statistics 19.0 Software (SPSS Inc, USA). One‐way and univariate analysis of variance (ANOVA) were applied to examine the effects of different treatments. The experiment was replicated twice for the GC‐MS and three times for others. Data are expressed as the mean ± standard deviation. Duncan's multiple range test was performed for post hoc multiple comparisons with the level of significance set at *p* < .05. The Pearson correlation coefficients, between oxidation stability index and free radical, were calculated using the bivariate analysis.

## 3 RESULTS AND DISCUSSION

### 3.1 Changes in peroxide value of infant formula powder with different ingredients

**Figure 1:**
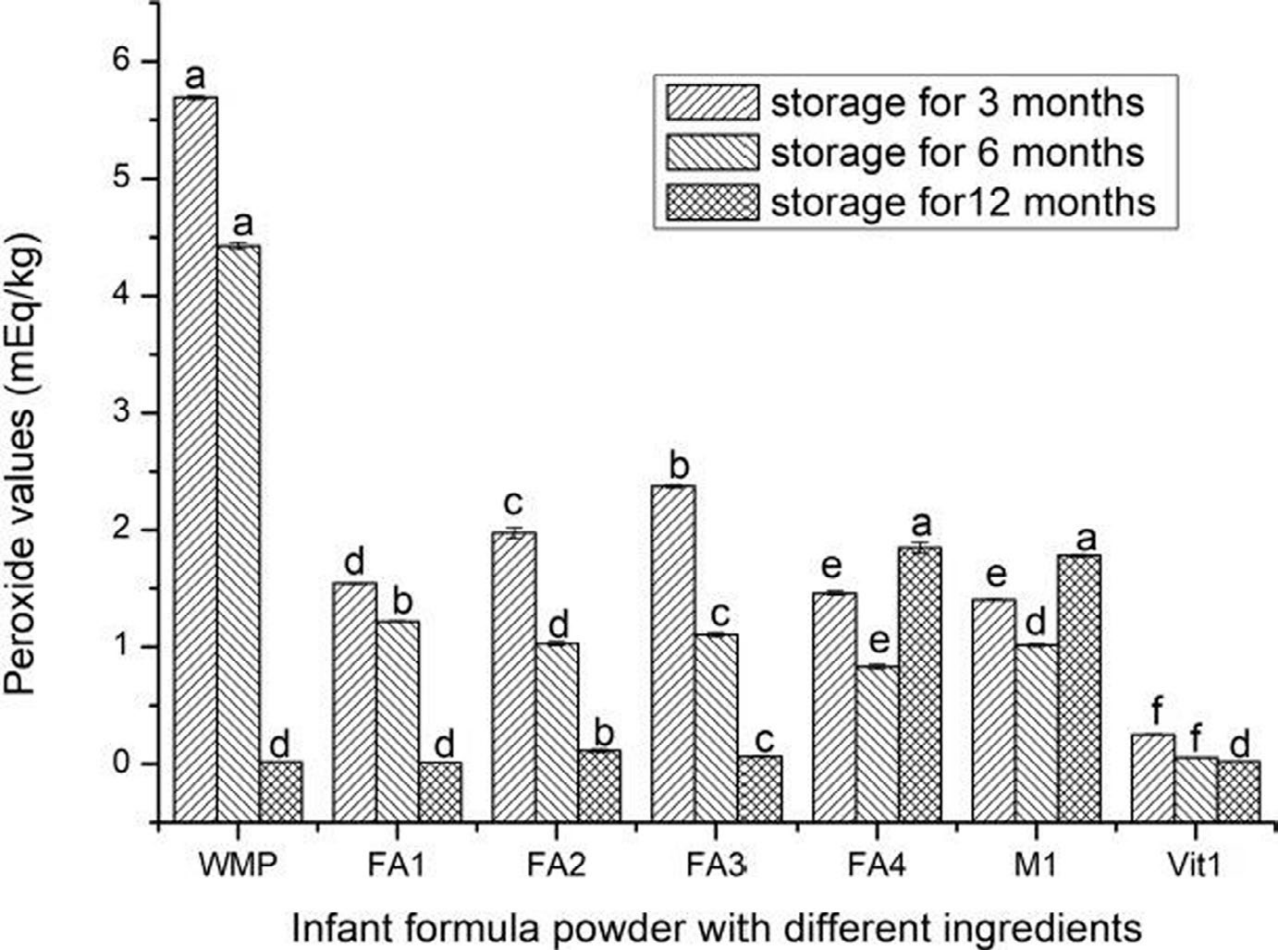
Peroxide values of infant formula powder with different ingredients during storage

Peroxide is the primary oxidation product produced during the lipid oxidation (Zou, 2002; Li et al., 2019). Figure 1 shows the change in peroxide value of infant formula powder with different ingredients during storage. When stored for 3 and 6 months, the content of peroxide value in WMP was the highest and lowest in Vit1. The results indicated the addition of vitamins could significantly reduce the peroxide value of infant formula powder with different ingredients (*p* < .05). During short‐term storage, the peroxide value of all the samples had the trend to decrease. At 12 months storage, the peroxide values of FA4 and M1 were 1.847 mEq/kg and 1.780 mEq/kg, respectively, which were higher than those at 6 months storage. This is consistent with the previous results, which indicated lipid peroxidation is high in infant formula powder enriched with polyunsaturated fatty acids (Almansa et al., 2013). On the whole, these results might be caused by the oxidation reactions of unsaturated fatty acids and metal ions during storage (Fumio et al., 1998; Smet et al., 2009).

### 3.2 Changes in TBA value of infant formula powder with different ingredients

**Figure 2:**
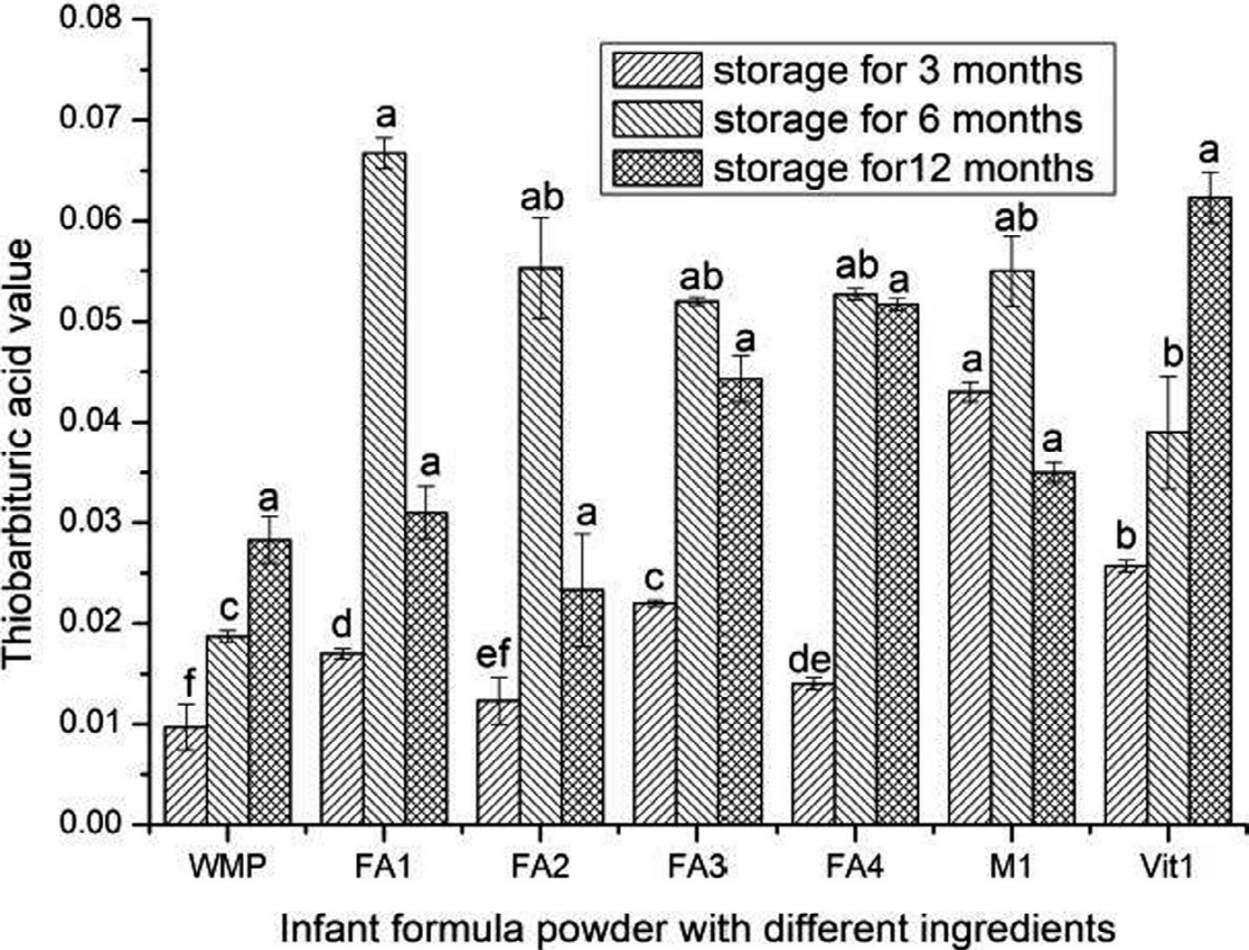
Thiobarbituric acid value of infant formula powder with different ingredients during storage

Thiobarbituric reactive substances are products of lipid peroxidation and are generated by the further degradation of lipid hydroperoxides. The TBA value is the main parameter to reflecting the further oxidation of milk fat (Guillen‐Sans & Guzman‐Chozas, 1995). Figure 2 shows the changes in TBA value of infant formula powder with different components. When the sample was stored for 3 months, the TBA value of WMP was the lowest and that of M1 was the highest. When stored for 6 months, there was no significant difference in TBA value among FA1, FA2, FA3, FA4, and M1 (*p* > .05). When the milk powder sample was stored for 3 and 6 months, the content of TBA in WMP was lower than that in infant formula powder, indicating that the addition of unsaturated fatty acids, metal ions, vitamins could increase the TBA value of milk powder during short‐term storage. Similar to this study, it had been proved that the oxidation of fat in DHA‐added milk powder was higher than that of fat in ordinary milk powder by determining TBA value (Joon‐Hwan & Choe, 1995; Stefania et al., 2015).

### 3.3 Free fat content of infant formula powder with different ingredients

**Figure 3:**
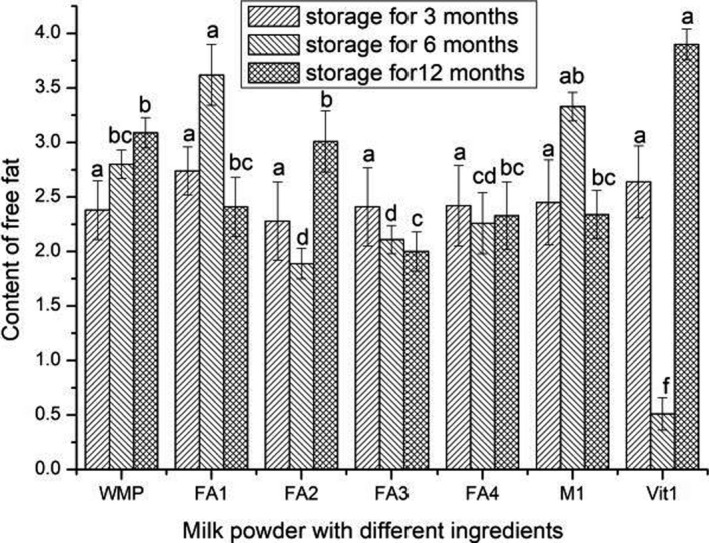
Content of free fat in infant formula powder with different ingredients during storage. The results with the same marking letters in the same form were not significantly different (*p* > .05)

The free fat content, an important index for evaluating the quality of milk powder, is considered to be the main reason for changes in oxidation flavors, the decrease in rehydration and the fluidity of milk powder in the drying industry (Marie‐Laure et al., 2007). Figure 3 shows the changes in free fat content of infant formula powder with different ingredients. It could be seen that there was no significant difference in the free fat of all samples after 3 months storage (*p* > .05). After 6 months of storage, the free fat content of FA1 and M1 was 3.62 g/100 g and 3.33 g/100 g, respectively. It indicated that the configuration of the base powder and the addition of metal ions could increase the free fat content of infant formula powder. There was no significant difference in the free fat content of FA2, FA3, and FA4 (*p* > .05). The free fat content of these samples with DHA and AA was lower than that of WMP and FA1. The result indicated that the addition of unsaturated fatty acids reduced the free fat content of infant formula powder with different ingredients. The free fat content of Vit1 was the lowest (0.51 g/100 g) among the samples (*p* < .05), which indicated that addition of vitamins could reduce the free fat content of infant formula powder after 6 months of storage. It increased to 3.90 g/100 g after 12 months of storage. These results indicated exist of vitamins could influence the oxidative stability of milk. Similar to this study, O’Brien and O’Connor (2011) reported that vitamins could provide antioxidant effect and prooxidant effect under different circumstances. Also, the crystallization lactose might trigger the release of entrapped fat from the infant formula powder (Juhi et al., 2020).

### 3.4 Color change of infant formula powder with different ingredients

Table 1 shows the color values (L and B) of infant formula powder with different ingredients during storage. Color value L of all samples had the trend to decrease, and it indicated that the brightness decreased. The results possibly caused by Maillard reaction and oxidation reaction (Wu et al., 2010). Among the infant formula powder with different ingredients, the color value L of FA1 was the highest (99.0) after 3 months of storage (*P *< .05). This might be due to the addition of lactose during the preparation of base milk powder, which increased the Maillard reaction. After 6 months of storage, there was no significant difference in color value L of all samples (*p* > .05). At 12 months of storage, the color values L of M1 and Vit1 were lower than that of FA1 (*P *< .05). Also, the color value L of Vit1 was the lowest (90.6) and there was a significant difference with other samples (*P < *0.05), which indicated that the addition of vitamins could accelerate the decrease of the brightness of infant formula powder during storage. This was probably related to the stability of vitamins in the infant formula powder (Chavez‐Servin et al., 2008). Color value B of WMP in different storage periods (18.8, 19.5, and 19.7, respectively) had high level; it decreased in infant formula powder with different ingredients, and there was no significant difference among the infant formula powder samples (*p* > .05).

**Table 1:** Color value L and Color value B in the infant formula powder with different ingredients during storage

Sample	Color value L			Color value B		
	3 months	6 months	12 months	3 months	6 months	12 months
WMP	94.3 ± 0.5^bA^	93.2 ± 0.1^aAB^	92.8 ± 0.2^aB^	18.8 ± 0.1^aB^	19.5 ± 0.1^aAB^	19.7 ± 0.3^aA^
FA1	99.0 ± 0.5^aA^	94.1 ± 0.1^aB^	93.5 ± 0.1^aB^	14.6 ± 0.3^bA^	14.1 ± 0.6^bA^	14.6 ± 0.1^bA^
FA2	95.3 ± 0.3^bA^	93.8 ± 0.2^aAB^	93.0 ± 0.2^aB^	14.3 ± 0.5^bA^	14.5 ± 1.0^bA^	14.5 ± 1.5^bA^
FA3	94.9 ± 0.3^bA^	94.2 ± 0.1^aAB^	92.3 ± 0.6^aB^	14.6 ± 0.4^bA^	14.6 ± 0.3^bA^	15.0 ± 0.1^bA^
FA4	95.4 ± 0.5^bA^	93.8 ± 0.8^aAB^	92.8 ± 0.6^aB^	14.2 ± 0.3^bA^	14.1 ± 0.4^bA^	15.0 ± 0.2^bA^
M1	95.4 ± 0.1^bA^	93.6 ± 0.2^aAB^	92.0 ± 0.1^abB^	13.8 ± 0.3^bA^	14.3 ± 0.1^bA^	14.9 ± 0.5^bA^
Vit1	94.9 ± 0.8^bA^	93.5 ± 1.4^aAB^	90.6 ± 0.1^bB^	14.3 ± 0.2^bA^	15.0 ± 0.4^bA^	15.8 ± 0.7^bA^

Note

Different lower‐case letters in the same column indicate significant differences between the samples (*p* < .05), and different upper‐case letters in the same row indicate significant differences between storage times (*p* < .05).

### 3.5 Change in the oxidized flavor compounds of infant formula powder

Aldehydes and ketones are the main volatile secondary oxidation products. Many studies have analyzed these volatiles to evaluate the oxidation of milk powder (Li, Zhang, Wang, Han 2013; Lloyd et al., 2009). The typical substances, hexanal, 2‐heptanone, and 2‐nonaone, were selected to reflect the oxidized flavor. Table 2 shows the content of the oxidized flavor compounds in infant formula powder during storage. The content of hexanal in different samples had the trend to increase. After the storage of 3 months and 6 months, the content of hexanal in M1 was the highest, which was 0.56 mg/L and 3.89 mg/L, respectively. The content of hexanal, 2‐heptanone, and 2‐nonaone in M1 was significantly higher than that of WMP and FA1 samples (*p* < .05), which indicated that the addition of metal ions could promote the formation of these oxidized flavor compounds during the short‐term storage. This was probably due to metal ions functioned as pro‐oxidants primarily by decomposing hydroperoxides to generate new reaction chains and influenced the oxidative stability (O’Brien & O’Connor, 2011). After the storage of 6 and 12 months, the contents of 2‐heptanone and 2‐nonaone in WMP were the highest. With the prolongation of storage time, the content of 2‐heptanone and 2‐nonaone in WMP and FA2 increased. The result indicated DHA could promote the formation of 2‐heptanone and 2‐nonaone in the infant formula powder during storage.

**Table 2:** Content of oxidized flavor compounds in the infant formula powder with different ingredients (mg/L)

Sample	Hexanal			2‐Heptanone			2‐Nonone		
	3 months	6 months	12 months	3 months	6 months	12 months	3 months	6 months	12 months
WMP	0.12^de^	0.51^c^	0.60^e^	5.13^a^	13.76^a^	20.47^a^	‐	2.35^a^	4.25^a^
FA1	0.09^e^	0.63^b^	0.81^d^	0.94^c^	6.43^d^	4.63^c^	0.07^d^	1.70^c^	0.92^c^
FA2	0.20^bc^	0.69^b^	2.33^a^	1.06^bc^	5.27^e^	15.18^b^	‐	1.00^f^	3.42^b^
FA3	0.14^de^	0.68^b^	0.75^d^	0.64^d^	7.13^c^	3.23^d^	‐	1.54^d^	0.85^c^
FA4	0.18^cd^	0.68^b^	0.70^de^	1.09^bc^	6.34^d^	0.90^e^	0.25^b^	1.27^e^	0.99^c^
M1	0.56^a^	3.89^a^	1.60^b^	1.30^b^	11.21^b^	4.64^c^	0.10^c^	2.17^b^	0.95^c^
Vit1	0.24^b^	0.70^b^	0.96^c^	1.02^c^	5.23^e^	4.64^c^	0.28^a^	1.19^e^	0.94^c^

Note

The results with the same superscript letters in each column were not significantly different (*p* > .05).

‐, The oxidized flavor compound had not been determined.

### 3.6 The free radicals in infant formula powder with different ingredients

A series of free radicals could produce by redox reactions during the storage of milk powder (Li et al., 2019). The free radicals of different milk powder stored for 6 months and 12 months were determined. The type and intensity of free radicals of different infant formula powder are shown in Table 3. It could be seen that the g value of free radical in WMP was 2.0060. The g value of FA1, FA2, and FA3 was 2.0048. As the two components of DHA and AA were added into the infant formula powder at the same time, the g value of FA4 was 2.0043. The g values of infant formula powder samples added metal ions and vitamins were 2.0052 and 2.0057, respectively. On the whole, the peak width and peak height of free radicals changed among the samples, which ranged 32.46–65.78 G and (3.116–7.766) × 10^5^, respectively.

**Table 3:** Type and intensity of free radicals in the infant formula powder with different ingredients during storage

Sample	G values		Peak width (G)		Peak height (*10^5^)	
	6 months	12 months	6 months	12 months	6 months	12 months
WMP	2.0060	2.0029	33.61 ± 2.97^c^	35.20 ± 0.74^c^	7.082 ± 0.008^b^	4.028 ± 0.083^a^
FA1	2.0048	2.1338	57.67 ± 3.78^ab^	133.60 ± 14.71^b^	4.468 ± 0.065^d^	1.947 ± 0.006^b^
FA2	2.0048	2.0017	32.46 ± 0.54^c^	48.33 ± 16.34^c^	3.116 ± 0.025^e^	1.331 ± 0.004^c^
FA3	2.0048	2.0028	65.78 ± 5.53^a^	38.58 ± 2.69^c^	4.426 ± 0.001^d^	1.171 ± 0.008^c^
FA4	2.0043	2.0032	44.30 ± 2.16^bc^	334.03 ± 0.01^a^	7.766 ± 0.320^a^	1.168 ± 0.093^c^
M1	2.0052	‐	46.59 ± 9.73^bc^	‐	5.314 ± 0.022^c^	‐
Vit1	2.0057	2.0018	51.56 ± 3.78^ab^	31.03 ± 0.70^c^	5.083 ± 0.099^c^	1.252 ± 0.045^c^

Note

The results with the same superscript letters in each column were not significantly different (*p* > .05).

‐, The free radicals had not been determined.

After the storage of 12 months, the *g* values of free radicals in different samples changed. Surprisingly, there was no free radical peak in M1 samples, which indicated that metal ions participated in the formation and quenching of free radicals in the samples. The range of *g* value among other samples was 2.0017–2.1338. The *g* value of FA1 was the highest. From the intensity of free radicals, the peak width of free radicals in FA1 and FA4 increased to 133.60 G and 334.03 G, respectively. At the same time, the peak height had a downward trend and it ranged (1.168–4.028) ×10^5^. The above results showed that the type and intensity of free radicals in infant formula powder changed with the components. Also, the existence of free radicals suggested that oxidation might be accelerated during the storage of milk powder (O’Brien & O’Connor, 2011; Stapelfeldt et al., 1997).

### 3.7 Correlation analysis of the index in infant formula powder during storage

During the storage of milk powder, the index related to oxidative stability changed differently. Free radical was an important indicator for evaluation the lipid oxidation of dairy products (Li et al., 2019; Stapelfeldt et al., 1997). Correlation analysis between oxidation stability index and free radical is calculated using the bivariate analysis (Table 4). It could be seen that when the infant formula powder was stored for 6 months, there was no significant correlation among the index of peroxide value, TBA value, free fat, color value L, color value B, hexanal, 2‐heptanone, and 2‐nonaone. When the infant formula powder was stored for 12 months, there was a significant correlation between the peroxide value and the peak width of free radicals (*p* = .920). The correlation coefficient was 0.908 between color value B and the peak width of free radicals. It was not significant between methyl ketone, TBA value, surface fat, and free radicals (*p* > .05). These results indicated that the index of peroxide value and color value B were significantly related to the existence of free radicals in the infant formula powder.

**Table 4:** Correlation coefficient between free radical and stability index of the infant formula powder

Stability index	Free radical intensity of the infant formula powder during storage			
	Peak width of 6 months	Peak height of 6 months	Peak width of 12 months	Peak height of 12 months
peroxide value	−0.465	0.419	0.920**	−0.295
TBA value	0.447	−0.495	−0.193	−0.346
Free fat	0.010	0.101	−0.433	0.184
color value L	0.672	−0.474	0.317	0.284
color value B	−0.488	0.427	−0.305	0.908*
hexanal	−0.008	0.027	−0.265	−0.320
2‐heptanone	−0.334	0.469	−0.524	0.761
2‐nonone	−0.075	0.388	−0.379	0.717

* Significant at 0.05 level (bilateral).

** Significant at 0.01 level (bilateral).

## 4 CONCLUSIONS

Different ingredients in infant formula powder exerted the profound influences on the progress of oxidation. The addition of metal ions, polyunsaturated fatty acids, and vitamins could reduce the peroxide value and increase the thiobarbituric acid value during the early stage of storage. The addition of metal ions could increase the free fat content of infant formula powder. While after adding vitamins, the content of free fat in infant formula powder decreased at the beginning of storage and then increased, which also could accelerate the decrease of the brightness of infant formula powder. Color value B of infant formula powder with different ingredients decreased during storage. The content of hexanal in different infant formula powder had the trend to increase. The addition of metal ions would promote the formation of hexanal, 2‐heptanone, and 2‐nonaone in the infant formula powder. The free radicals were not inconsistent during the storage. The types and intensities of free radicals changed with the composition. The peroxide value and color value B had high correlations with the existence of free radicals in the infant formula powder. More attention should be paid to study on the investigation of improving the quality and prolonging storage period of infant formula powder.
